# Management of Patients Presenting with Acute Subdural Hematoma due to Ruptured Intracranial Aneurysm

**DOI:** 10.1155/2012/753596

**Published:** 2012-03-01

**Authors:** Serge Marbacher, Ottavio Tomasi, Javier Fandino

**Affiliations:** Department of Neurosurgery, Kantonsspital Aarau, 5001 Aarau, Switzerland

## Abstract

Acute subdural hematoma is a rare presentation of ruptured aneurysms. The rarity of the disease makes it difficult to establish reliable clinical guidelines. Many patients present comatose and differential diagnosis is complicated due to aneurysm rupture results in or mimics traumatic brain injury. Fast decision-making is required to treat this life-threatening condition. Determining initial diagnostic studies, as well as making treatment decisions, can be complicated by rapid deterioration of the patient, and the mixture of symptoms due to the subarachnoid hemorrhage or mass effect of the hematoma. This paper reviews initial clinical and radiological findings, diagnostic approaches, treatment modalities, and outcome of patients presenting with aneurysmal subarachnoid hemorrhage complicated by acute subdural hematoma. Clinical strategies used by several authors over the past 20 years are discussed and summarized in a proposed treatment flowchart.

## 1. Introduction

Rupture of a cerebral aneurysm normally results in subarachnoid hemorrhage (SAH) and is often complicated by intracerebral hematoma (ICH), but only on rare occasions does it cause acute subdural hematoma (aSDH) [1]. Diagnosis of aneurysmal SAH can be difficult in comatose patients in whom loss of conscious due to aneurysm rupture results in or mimics a traumatic brain injury [2]. Determining a differential diagnosis and treatment modalities can further be complicated by the rapid clinical course and the mixture of symptoms due to the ruptured aneurysm and the mass effect of the hematoma.

Rapid decision making is required to treat this life-threatening condition. The majority of patients with aneurysmal SAH and coincidental acute subdural bleeding present in a severe clinical condition, and immediate surgical management is required [2–4]. Decisions to be made include whether preoperative diagnostic studies should precede surgery and whether obliteration of the aneurysm should be performed during hematoma evacuation or in a separate delayed intervention after resuscitation procedures.

The incidence of combined SAH and aSDH varies from 0.5% [5, 6] to 10% [7] in clinical series. The rarity of aneurysmal aSDH makes it difficult to design reliable clinical treatment guidelines. Large systematic series do not exist, and thus treatment decisions are mainly based on personal experience. The aim of this review is to propose a management flow chart and protocol based on published experience with such cases over the past two decades. 

## 2. Materials and Methods

### 2.1. Search Strategy

The literature was screened for case studies of acute subdural hematoma secondary to ruptured intracranial aneurysm. Articles for this review were identified by MEDLINE PubMed database searches of the literature from January 1990 through December 2009 using the terms “acute subdural hematoma,” “subarachnoid hemorrhage,” and “cerebral aneurysm” (by using the Boolean operator AND) (Table 1). The senior author independently assessed the reproducibility of the search strategy on August 30, 2010, two days after the first author's search. Cross-references were checked in each eligible article.

### 2.2. Selection Criteria

 Articles were excluded based on title and abstract because they (i) were not written in the English language, (ii) were technical notes or laboratory investigations, or (iii) were not peer-reviewed/original studies. The remaining articles were selected for inclusion if the patients were adults and the single cases or case series provided detailed descriptions of clinical characteristics and patient management.

### 2.3. Data Acquisition

From selected cases, we extracted the following characteristics and recorded them in a data sheet: age; gender; initial clinical findings, including Glasgow Coma Scale (GCS) [8] score, clinical SAH grade based on the Hunt and Hess (H&H) [9, 10], and the World Federation of Neurological Surgeons (WFNS) [11] classifications; presence of major (aphasia, hemiparesis, or hemiplegia) and minor (cranial nerve palsies) focal neurological deficits, hemodynamic situation at the time of admission; radiological assessment, including computed tomography (CT) scan, CT angiography (CTA), magnetic resonance imaging (MRI), MR angiography (MRA), and digital subtraction angiography (DSA); additional presence of SAH, intracerebral hematoma (ICH); side and size of aSDH and associated midline shift; aneurysm size and location; case management; outcome according to the Glasgow Outcome Scale (GOS), modified Rankin Score (mRS), and Barthel index (BI).

## 3. Results

**Figure 1:**
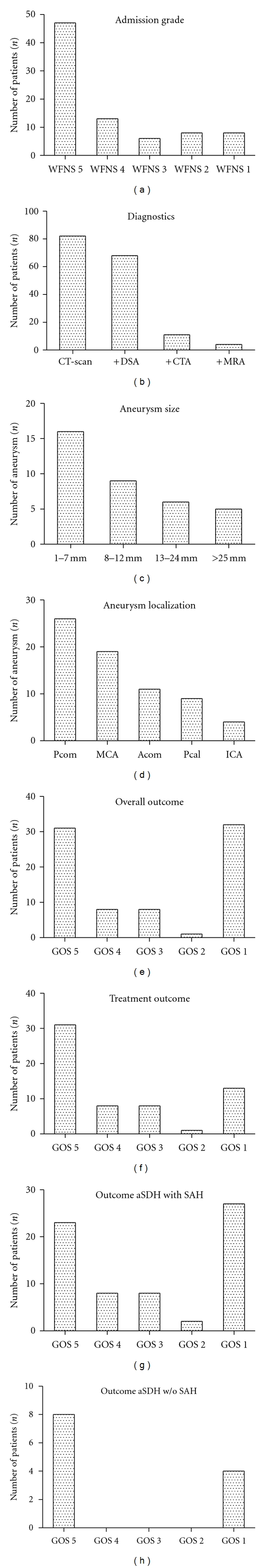
Data analysis of 82 cases of aneurysmal aSDH*. *Abbreviations: WFNS = World Federation of Neurological Surgeons; CT = computed tomography; DSA = digital subtraction angiography; CTA = CT angiography; MRA = Magnetic resonance angiography; mm = millimeter; Pcom = posterior communicating artery; MCA = middle cerebral artery; Acom = anterior communicating artery; Pcal = pericallosal artery; ICA = internal carotid artery; GOS = Glasgow Outcome Scale.

The initial search retrieved 85 publications which matched the terms “cerebral aneurysm” AND “subarachnoid hemorrhage” AND “acute subdural hematoma.” 59 publications were excluded after screening of titles and abstracts. This left 26 articles potentially eligible for detailed evaluation. Six articles were not included as they did not match the selection criteria. The remaining 20 articles including 82 cases underwent detailed analysis [2–4, 12–14, 16–26, 28–30]. Characteristics of the 82 cases are summarized in Table 2. Graphs displaying the analyzed data appear in Figure 1.

### 3.1. Initial Clinical Findings

Most of the patients were admitted with the worst initial clinical SAH grades and with signs of uncal herniation. The distribution according to the WFNS was grade 5 (*n* = 46, 57.3%), grade 4 (*n* = 14, 17.1%), grade 3 (*n* = 6, 7.3%), grade 2 (*n* = 8, 9.8%), and grade 1 (*n* = 8, 9.8%). At admission, signs of uncal herniation, major focal neurological deficits, and minor focal neurological deficits were present in 35 (42.7%), eight (9.8%), and six (7.3%) patients, respectively. Fourteen (17.1%) patients presented in an unstable cardiopulmonary condition (e.g., ventricular arrhythmia, acute heart failure, and sudden pulmonary edema) at the time of admission. Four (4.9%) patients died during resuscitation. One (1.2%) patient was reported to have had prolonged hypoxia.

### 3.2. Diagnostic Approaches and Radiological Findings

**Figure 2:**
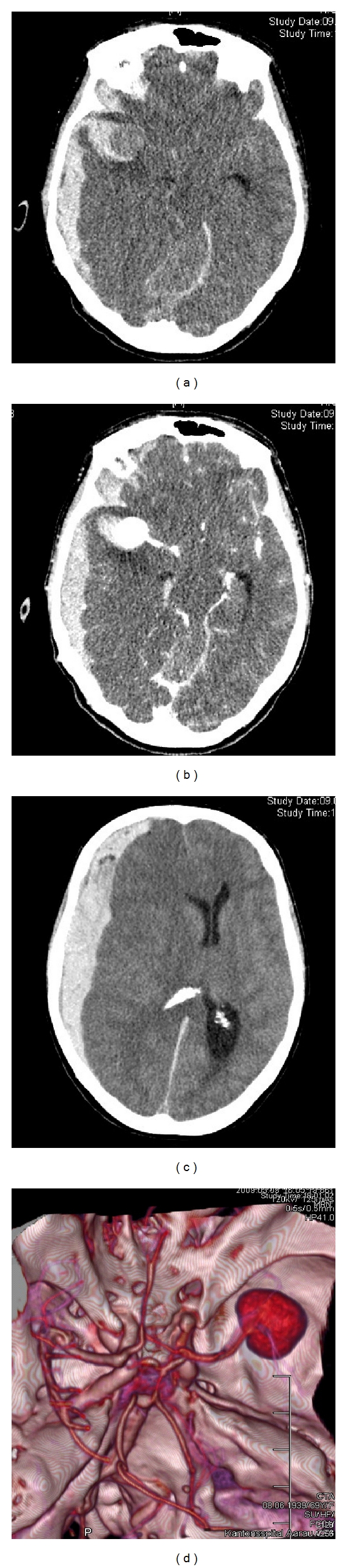
Illustrative case: Panels (a–d) display axial CT scans with 3D reconstructions showing a right acute subdural hematoma with midline shift after rupture of a giant aneurysm located in the right middle cerebral artery. Panels (a) and (b): noncontrast-enhanced and contrast-enhanced axial CT scan, demonstrating a large aneurysm in the right silvian fissure with surrounding SAH, right-sided aSDH, and uncal herniation. Panel (c) shows a marked midline shift due to the mass effect of the aSDH. Panel (d) depicts the aneurysm with outgoing vessels.

For all patients, the first radiological assessment was a CT scan (*n* = 82, 100%). 68 (82.9%) patients underwent additional DSA, and 11 (13.4%) underwent additional CTA (Figure 2). Four (4.9%) patients underwent MRA prior to surgery. SAH was detected on initial CT scan in 68 (82.9%) patients. There were 13 (15.9%) cases of pure aSDH without associated SAH. 28 (34.1%) patients presented with additional ICH. In 24 (29.3%) patients, the size of the aSDH was reported (mean ± SD: 9.6 ± 3.5, range: 5–20 mm). A total of 30 (36.6%) patients were reported as presenting with midline shift associated with aSDH (mean ± SD: 9.1 ± 4.0, range: 4–23 mm). All but six cases (7.3%) of aSDH were documented ipsilateral to the side of the aneurysm. Two cases presented with bilateral aSDH. Aneurysm size was reported in 37 (45.1%) patients (mean ± SD: 11.4 ± 8.1, range: 1.5–30 mm).

### 3.3. Aneurysm Localization

In most of the cases, the aneurysm was located in the posterior communicating artery (Pcom) (*n* = 39, 46.6%). The rest of the aneurysms were located in the middle cerebral artery (MCA) (*n* = 20, 23.2%), the anterior communicating artery (Acom) (*n* = 11, 13.4%), the pericallosal artery (Pcal) (*n* = 8, 9.8%), or the internal carotid artery ICA (*n* = 4, 4.9%).

### 3.4. Treatment Strategies

The treatment strategies included urgent hematoma evacuation (*n* = 59, 72%), surgical aneurysm obliteration in the same procedure as urgent hematoma evacuation (*n* = 41, 50%), delayed clipping (*n* = 10, 12.2%), and delayed coiling (*n* = 6, 7.3%). Eighteen patients (22%) died during resuscitation or did not meet the criteria for undergoing any of the invasive procedures due to cardiopulmonary instability. A total of six (7.3%) patients underwent external ventricular drainage, and ten (12.2%) patients were treated with hyperosmolar therapy.

### 3.5. Outcome

Half of the patients were reported to have favorable outcomes (GOS 5 and GOS 4, *n* = 39, 47.6%). Poor outcome (GOS 3 and GOS 2) was reported in nine (11%) patients. 32 patients (26.6%) had fatal outcomes (GOS 1). Overall distribution according to the GOS was GOS 5 (*n* = 31, 37.8%), GOS 4 (*n* = 8, 9.8%), GOS 3 (*n* = 8, 9.8%), GOS 2 (*n* = 1, 1.2%), and GOS 1 (*n* = 32, 39%). In 19 (23.2%) out of 32 patients with fatal outcome (GOS 1), the critical status at admission did not allow any surgical or endovascular intervention. Four (4.9%) patients died during resuscitation, two (2.4%) patients died immediately after diagnosis, and one (1.2%) patient received no further therapy as a result of prolonged hypoxia before admission. Most of the 63 patients who met the criteria for invasive treatment achieved good outcomes (GOS 5 and GOS 4, *n* = 39, 69.9%). The distribution of these patients by treatment outcome according to the GOS was GOS 5 (*n* = 31, 49.2%), GOS 4 (*n* = 8, 12.7%), GOS 3 (*n* = 8, 12.7%), GOS 2 (*n* = 1, 1.6%), and GOS 1 (*n* = 13, 20.6%). Patients who suffered aneurysmal aSDH without SAH demonstrated better outcomes (GOS 5, *n* = 9, 69.2%; GOS 1, *n* = 5, 38.5%) than patients who presented with aneurysmal aSDH and SAH (GOS 5, *n* = 22, 31.4%; GOS 4, *n* = 8, 11.4%; GOS 3, *n* = 8, 11.4%; GOS 2, *n* = 1, 1.4%; GOS 1, *n* = 27, 38.6%).

### 3.6. Outcome Stratified by Therapeutic Strategies (Table 3)

All patients presenting in good clinical condition without rapid neurological deterioration (*n* = 15) demonstrated good outcomes (GOS 5 and GOS 4). These outcomes were favorable irrespective of whether hematoma evacuation and aneurysm obliteration were immediate (*n* = 10) or delayed (*n* = 5). However, patients with rapidly deteriorating levels of consciousness (including signs of brain herniation) and urgent (<24 h) intervention had a higher likelihood of good outcomes (GOS 5 and GOS 4) than patients with rapid deterioration who had undergone delayed (24 h) treatment (64% versus 25%).

## 4. Discussion

This meta-analysis of 82 reported cases presenting with aneurysmal aSDH and rapid neurological deterioration revealed that urgent surgical decompression and immediate occlusion of the aneurysm seem to be an acceptable treatment strategy in order to achieve better outcome (GOS 5 and GOS 4 = 64%). Good outcomes are found in patients maintaining stable neurological condition irrespective of whether intervention was immediate or delayed (GOS 5 = 100%). Patients with pure aSDH due to a ruptured aneurysm demonstrated better outcomes than patients who suffered aneurysmal aSDH associated with SAH. Patients in unstable cardiopulmonary condition, with unstable blood pressure and serious ventricular arrhythmias, have the highest risk of unfavorable outcomes. All patients who did not meet the criteria for invasive treatment had fatal outcomes.

Poor clinical presentation per se is not associated with worse outcome. However, the combination of marginal cardiac output and reduced cerebral perfusion and cerebral blood flow due to the mass effect [31] during the acute phase of SAH [32] is likely to result in poor final outcome. Patients presenting in such condition do not meet the criteria for urgent hematoma evacuation, which additionally worsens the likelihood of favorable outcome (GOS 5 and GOS 4 = 25%). Patients in stable hemodynamic condition are suitable for rapid surgical decompression and maximal medical treatment and have a higher chance of recovering in good neurological condition (GOS 5 and GOS 4 = 64%) despite severe SAH and poor initial GCS admission scores. Two-thirds of all patients with either poor grade SAH or traumatic aSDH usually do not survive, and functional outcome is rare [33–35]. The good recovery of patients with aneurysmal aSDH might be explained by the space-occupying effect of the hematoma, which mimics a worse clinical situation and does not reflect vital brain destruction.

Pure aSDH due to ruptured intracranial aneurysm is extremely rare. Only 20 cases have been reported so far, including 14 cases during the last two decades [16]. In most cases of aneurysmal aSDH, the history will distinguish a traumatic from a spontaneous cause [1]. However, the absence of hematomas and subarachnoid blood collections related to common aneurysm sites can impede the diagnosis. The finding that pure aneurysmal aSDH results in better outcome than aSDH with SAH may be explained by the fact that these patients less frequently have complications (delayed cerebral vasospasm and hydrocephalus).

Due to the rarity of the disease, no guidelines have been established. In most reports, patients have bad clinical features on admission, often presenting in a comatose state with pupillary abnormalities. Fast decision making is mandatory. Determining a differential diagnosis, as well as treatment modalities, can be complicated by the rapid clinical course and the mixture of symptoms due to the ruptured aneurysm or mass effect of the hematoma.

To address the lack of guidelines, we developed a flowchart for treatment of patients with aSDH. However, the evidence for the proposed treatment flowchart comes from case series and case reports with relatively small sample sizes. Therefore, the estimation of effects is imprecise, and clinical recommendations included in the management protocol are weak [36, 37].

**Figure 3:**
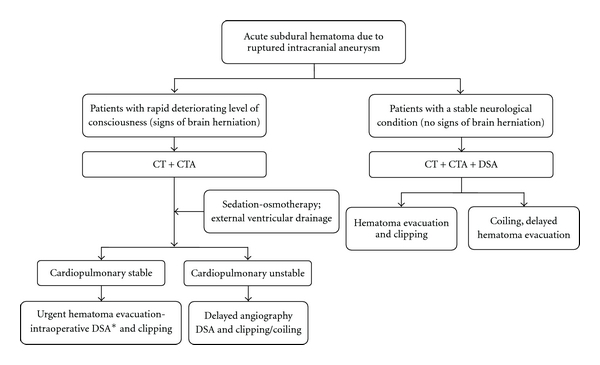
Illustrative schematic diagram of the protocol (management algorithm) for diagnosis and treatment of aneurysmal acute subdural hematoma. CT = computed tomography. CTA = CT angiography. DSA = digital subtraction angiography. * = if available.

In patients who are in good neurological condition at the time of admission, management may proceed in a standard manner (Figure 3, left side of the flowchart). After initial CT and CTA examination, DSA is the diagnostic modality of choice to verify the angioarchitecture of the aneurysm. If the aneurysm is suitable for endovascular obliteration and the aSDH remains clinically insignificant, the aneurysm can be occluded during the same procedure [4]. If a decision is made to occlude the aneurysm surgically, DSA provides relevant anatomical information and guidance in determining a clipping strategy and surgical approach.

 For the management of patients who are in a coma or whose level of consciousness is deteriorating rapidly, the choice of initial diagnostics is more demanding, and management decisions become difficult (Figure 3, right side of the flowchart). The aSDH may be the major determinant of neurological grade, and prompt hematoma evacuation may be life saving. At the minimum, neuroradiological investigations should consist of an emergency CT and CTA to visualize potential bleeding sources. Emergency treatment modalities such as maximal sedation, osmotherapy, and external ventricular drainage to reverse signs of brain herniation should be performed as quickly as possible. In these cases, the emergency situation forces the neurosurgeon to postpone DSA.

Intraoperative DSA would allow safe and complete aneurysm occlusion to be carried out at the same time as urgent hematoma evacuation [38, 39]. Patients would be spared a second procedure. However, Westermaier et al. [4] recently presented four patients who underwent separate delayed endovascular coiling after decompression and hematoma evacuation. Despite good neurological recovery in three of these four patients, subjecting patients to two separate procedures rather than clipping at the same time as hematoma removal remains controversial. Patients who present in unstable cardiopulmonary conditions cannot be operated on immediately. It seems that this subgroup of patients is exceptionally at risk of poor outcome. Withholding aggressive therapy in poor-grade patients in order to prevent vegetative survival is highly controversial and cannot be recommended.

## 5. Conclusion

Due to the rarity of aneurysmal aSDH, it remains difficult to define a comprehensive management protocol. In patients with poor neurological grade at admission and rapidly deteriorating levels of consciousness, urgent surgical decompression and immediate aneurysm obliteration result in favorable outcome (GOS 5 and GOS 4; 64%). Delay of immediate treatment in patients with rapidly deteriorating neurological conditions decreases the likelihood of a favorable outcome (GOS 5 and GOS 4; 25%). Good outcomes are observed in patients maintaining stable neurological condition irrespective of whether the intervention was immediate or delayed (GOS 5; 100%). Overall outcome of patients who suffered aneurysmal aSDH without SAH proved to be better (GOS 5, 69.2%) than the outcome of patients who presented with aneurysmal aSDH and SAH (GOS 5; 31.4%).

##  Conflict of Interests

The authors are solely responsible for the design and conduct of the presented study and report no conflict of interests. No funds were or will be received for this study.

## Figures and Tables

**Table 1:** Search Strategy*.

Search number	Process description	Results
	(“key words”)	(no. of articles)
no. 1	Search “cerebral aneurysm”	**22944**
no. 2	Search “subarachnoid hemorrhage”	**17883**
no. 3	Search “subdural hematoma”	**7732**
no. 4	Search #1 AND #2 AND #3	**155**
no. 5	Search “01/1990–12/2009” AND #4	**85**

*All searches for this study were performed on August 28, 2010, by the first author and verified by the second author on August 30, 2010. The publication date limits were set to January 1990–December 2009.

**Table 2:** Patient characteristics*.

Series/year of publication	Case no.	Age/sex	Initial clinical findings	Initial diagnostics	SAH	ICH	Side of aSDH	Size of aSDH	MLS	Location of aneurysm	Size of aneurysm	Management (hours from ictus)	Outcome
Watanabe et al. [30]/1991	1	51/m	WFNS 5, GCS 4, decerebrate posture, bilaterally dilated fixed pupils ataxic breath	CT scan	No	No	Lt	—	—	Lt Pcal (ACA)	—	Emergency craniectomy and hematoma evacuation (1 h)	Deceased, GOS 1, mRS 6

Watanabe et al. [30]/1991	2	72/f	WFNS 4, GCS 12, right hemiparesis	CT scan, DSA	Yes	No	Lt	—	—	Rt Pcal (ACA)	—	Clipping (on day 15)	Returned to normal daily life, GOS 5, mRS 1

Watanabe et al. [30]/1991	3	74/f	WFNS 5, GCS 4, decerebrate posture, ataxic breath, dilation of the left pupil	CT scan, DSA failed	Yes	No	Rt	—	—	Lt Pcal (ACA) (found at autopsy)	—	Inoperable	Deceased (3 days after onset), GOS 1, mRS 6

Kamiya et al. [5]/1991	4	67/f	H&H IV, paresis	CT scan, DSA	Yes	No	—	—	—	MCA	30 mm	Craniotomy, hematoma evacuation, and immediate clipping	Vegetative state, GOS 2, mRS 5

Kamiya et al. [5]/1991	5	50/f	H&H III	CT scan, DSA	Yes	Yes	—	—	—	ICA	28 mm	Inoperable because of rerupture on admission	Decubitus and pneumonia, deceased, GOS 1, mRs 6

Kamiya et al. [5]/1991	6	67/f	H&H V, paresis	CT scan, DSA	Yes	Yes	—	—	—	MCA	4 mm	Inoperable	Deceased (on arrival), GOS 1, mRs 6

Kamiya et al. [5]/1991	7	52/f	H&H V	CT scan, DSA	Yes	Yes	—	—	—	Not detected	—	Inoperable	Deceased (on arrival), GOS 1, mRs 6

Kamiya et al. [5]/1991	8	69/f	H&H II	CT scan, DSA	Yes	Yes	—	—	—	Acom	27 mm	Inoperable because of severe spasm on admission	Deceased, GOS 1, mRs 6

Kamiya et al. [5]/1991	9	63/f	H&H II	CT scan, DSA	Yes	No	—	—	—	MCA	4 mm	Craniotomy, hematoma evacuation, and immediate clipping	Good recovery, GOS 5, mRs 1

Kamiya et al. [5]/1991	10	73/m	H&H IV, paresis	CT scan, DSA	Yes	No	—	—	—	Acom	7 mm	Clinical deterioration, no operation impossible	Deceased, GOS 1, mRs 6

Kamiya et al. [5]/1991	11	64/m	H&H V, preoperative rerupture, cardiac failure	CT scan, DSA failed	Yes	No	—	—	—	Not detected	—	Inoperable	Deceased (nonfilling state DSA), GOS 1, mRs 6

Kamiya et al. [5]/1991	12	72/f	H&H IV, paresis	CT scan, DSA	Yes	Yes	—	—	—	Distal ACA	4 mm	Hematoma evacuation and immediate clipping	Good recovery, GOS 5, mRs 1

Kamiya et al. [5]/1991	13	70/m	H&H IV, paresis	CT scan, DSA	Yes	Yes	—	—	—	MCA	6 mm	Hematoma evacuation and immediate clipping	Good recovery, GOS 5, mRs 1

Kamiya et al. [5]/1991	14	72/f	H&H V	CT scan, DSA	Yes	Yes	—	—	—	ICA	4 mm	Inoperable	Deceased (day 1), GOS 1, mRs 6

Kamiya et al. [5]/1991	15	59/m	H&H V	CT scan, DSA	Yes	Yes	—	—	—	MCA	22 mm	Inoperable	Deceased (day 2), GOS 1, mRs 6

Kamiya et al. [5]/1991	16	39/f	WFNS 5, GCS 4, decerebrate posturing, dilation of the right pupil	CT scan, DSA	Yes	Yes	—	—	Moderate to marked	Distal ACA	3 mm	Hematoma evacuation and immediate clipping	Good recovery, GOS 5, mRs 1

Kamiya et al. [5]/1991	17	71/f	H&H III	CT scan, DSA	Yes	Yes	—	—	—	Acom	11 mm	Hematoma evacuation and immediate clipping	Good recovery, GOS 5, mRs 1

Rusyniak et al. [12]/1992	19	74/f	WFNS 5, GCS 4, decerebrate posturing, bilaterally miotic pupils	CT scan, CTA	Yes	Yes	Rt	—	—	Rt ICA-Pcom	—	Hematoma evacuation, immediate clipping	Complete recovery, GOS 5, mRS 1

Ragland et al. [13]/1993	20	27/m	GCS 5 right pupil nonreactive left mydriasis	CT scan, DSA	No	no	Rt	—	Moderate to marked	Acom	20 mm	Hematoma evacuation, Maximal medical treatment	Deceased, GOS 1, mRS 6

O'Sullivan et al. [3]/1994	21	32/m	WFNS 5, GCS 4, bilaterally fixed pupils, hypertensive with bradycardia	CT scan	Yes	—	Lt	—	—	Lt ICA-Pcom	12 mm	Mannitol, without effect on pupillary response (3 h), died before decompression	Deceased, GOS 1, mRS 6

O'Sullivan et al. [3]/1994	22	48/f	WFNS 5, GCS 4, dilated unreactive pupils, unstable cardiopulmonary situation	CT scan, DSA	Yes	Yes	Rt	—	—	Rt ICA-Pcom	15 mm	Manitol, without effect on pupillary response, hematoma evacuation, and clipping of the aneurysm (7 h)	Deceased, GOS 1, mRS 6

O'Sullivan et al. [3]/1994	23	36/f	WFNS 5, GCS 3, bilaterally fixed pupils	CT scan, DSA	Yes	Yes	Rt	—	—	Rt MCA	12 mm	Hematoma evacuation (4 h) and delayed clipping (day 4)	Residual mild left hemiparesis, returned to work as a teacher, GOS 4, mRS 3

O'Sullivan et al. [3]/1994	24	63/f	WFNS 5, GCS 3, dilated unreactive pupils	CT scan, DSA	Yes	—	Rt	—	—	Rt ICA-Pcom	20 mm	Hematoma evacuation (4 h) and delayed clipping (day 7)	Full recovery, returned to normal lifestyle, GOS 5, mRS 1

O'Sullivan et al. [3]/1994	25	62/f	WFNS 3, GCS 14, mild left hemiparesis	CT scan, DSA	Yes	No	Rt	20 mm	—	Rt ICA-Pcom	4 mm	Hematoma evacuation and immediate clipping	Uneventful recovery, returned to normal lifestyle, GOS 5, mRS 1

Nowak et al. [14]/1995	26	52/f	WFNS 5, GCS 3, dilated unreactive pupils, hypertensive crisis (systolic BP 280 mmHg)	CT scan	Yes	No	Rt	—	—	Rt Pcal (ACA)	—	Manitol, emergency hematoma evacuation	Deceased, GOS 1, mRS 6

Nowak et al. [14]/1995	27	45/f	WFNS 1, GCS 15, disturbances of vision	CT scan, DSA	Yes	Yes	Rt	10 mm	—	Rt MCA	—	Hematoma evacuation and clipping (day 1)	Full recovery, returned to normal lifestyle, GOS 5, mRS 1

Nowak et al. [14]/1995	28	49/f	WFNS 5, GCS 3, mild left-sided hemiparesis	CT scan	Yes	—	Rt	—	Marked	Rt MCA	>25 mm	Emergency hematoma evacuation with gluing of the aneurysm	Deceased, GOS 1, mRS 6 (rebleeding)

Nowak et al. [14]/1995	29	63/m	WFNS 5, GCS < 6, right dilated pupil	CT scan, DSA	Yes	—	Rt	—	—	Rt MCA	10 mm	Immediate hematoma evacuation and delayed clipping (week 5)	Full recovery, no serious neurological deficits, GOS 5, mRS 1

Ishibashi et al. [15]/1997	30	54/f	WFNS 1, GCS 15, no neurological deficit	CT scan, DSA	No	No	Lt	—	—	Lt ICA-PCom	—	Craniotomy, hematoma evacuation, and immediate clipping (<24 h)	No neurological deficit, return to normal life, GOS 5, mRS 1

Nonaka et al. [16]/2000	31	52/f	GCS 4, decerebrate rigidity, and left oculomotor paresis	CT scan, DSA	No	No	Lt	—	Moderate to marked	Lt ICA-PCom	10 mm	Craniotomy, hematoma evacuation, and immediate clipping (>24 h)	Full recovery, no neurological deficits, GOS 5, mRS 1

Inamasu et al. [17]/2002	32	68/m	WFNS 2, GCS 14, H&H II	CT scan, DSA	Yes	No	—	<25 cc	<5 mm	Acom	—	Craniotomy, hematoma evacuation, and immediate clipping (6 h)	Good recovery, GOS 5, mRs 1

Inamasu et al. [17]/2002	33	61/f	WFNS 4, GCS 10, H&H IV	CT scan, DSA	Yes	Yes	—	<25 cc	<5 mm	Rt MCA	—	Craniotomy, hematoma evacuation, and immediate clipping (6 h)	Good recovery, GOS 5, mRs 1

Inamasu et al. [17]/2002	34	75/f	WFNS 4, GCS 11, H&H IV	CT scan, DSA	Yes	Yes	—	<25 cc	<5 mm	Lt MCA	—	Craniotomy, hematoma evacuation, and immediate clipping (6 h)	Severe disability, GOS 3, mRS 5

Inamasu et al. [17]/2002	35	28/f	WFNS 5, GCS 5, H&H IV	CT scan,	No	No	Rt	<25 cc	>10 mm	Lt ICA-Pcom (autopsy)	—	Craniectomy and hematoma evacuation	Deceased (5 days after admission), GOS 1, mRS 6

Inamasu et al. [17]/2002	36	53/f	WFNS 5, GCS 4, H&H V, bilaterally dilated pupils	CT scan, DSA	Yes	No	Rt	<25 cc	>10 mm	Rt ICA-Pcom	—	Craniotomy, hematoma evacuation, and clipping	Deceased (3 days after admission due to severe postoperative brain swelling), GOS 1, mRS 6

Inamasu et al. [17]/2002	37	72/f	WFNS 5, GCS 4, H&H V	CT scan,	Yes	No	—	<25 cc	>10 mm	Lt ICA-Pcom (autopsy)	—	Infusions of manitol, burr hole	Deceased, GOS 1, mRS 6

Inamasu et al. [17]/2002	38	53/m	WFNS 5, GCS 5, H&H V	CT scan	Yes	No	—	<25 cc	>10 mm	Unknown	—	Infusions of manitol, burr hole	Deceased, GOS 1, mRS 6

Inamasu et al. [17]/2002	39	47/f	WFNS 5, GCS 4, H&H V	CT scan	Yes	No	—	<25 cc	>10 mm	Unknown	—	Infusions of manitol, burr hole	Deceased, GOS 1, mRS 6

Inamasu et al. [17]/2002	40	70/f	WFNS 5, GCS 4, H&H V	CT scan	Yes	Yes	—	<25 cc	>10 mm	Unknown	—	No response to manitol infusion, conservative treatment	Deceased, GOS 1, mRS 6

Inamasu et al. [17]/2002	41	81/f	WFNS 5, GCS 4, H&H V	CT scan	Yes	No	—	<25 cc	>10 mm	Unknown	—	No response to manitol infusion, conservative treatment	Deceased, GOS 1, mRS 6

Inamasu et al. [17]/2002	42	55/m	WFNS 5, GCS 3, H&H V	CT scan	Yes	No	—	<25 cc	>10 mm	Unknown	—	No response to manitol infusion, conservative treatment	Deceased, GOS 1, mRS 6

Inamasu et al. [17]/2002	43	49/m	WFNS 5, GCS 3, H&H V	CT scan	Yes	No	—	<25 cc	>10 mm	Unknown	—	No response to manitol infusion, conservative treatment	Deceased, GOS 1, mRS 6

Gelabert-Gonzalez et al. [18]/2004	44	68/f	WFNS 5, GCS 4, fixed pupils	CT scan, DSA	Yes	No	Lt	—	—	Lt ICA-Pcom	—	Hematoma evacuation and immediate clipping (4 h)	Mild right-sided hemiparesis, GOS 4, mRS 2

Gelabert-Gonzalez et al. [18]/2004	45	64/f	WFNS 4, GCS 9, dilation of the right pupil	CT scan, CTA	Yes	—	Rt	—	Marked	Lt ICA-Pcom	—	Hematoma evacuation and immediate clipping (28 h)	Full recovery, neurologically intact, GOS 5, mRS 1

Gelabert-Gonzalez et al. [18]/2004	46	41/f	WFNS 5, GCS 4, right oculomotor paresis	CT scan, DSA	Yes	Yes	Lt	—	Marked	Lt ICA-Pcom	—	Hematoma evacuation and immediate clipping (5 h)	Deceased, GOS 1, mRS 6

Gelabert-Gonzalez et al. [18]/2004	47	59/f	WFNS 5, GCS 6, bilaterally fixed pupils	CT scan, DSA	Yes	No	Rt	—	—	Rt ICA	3 mm	Hematoma evacuation and immediate clipping (9 h)	Deceased, GOS 1, mRS 6

Krishnaney et al. [19]/2004	48	42/f	WFNS 2, GCS 14	CT scan, MRI, MRA, DSA	No	No	Bilateral	—	—	Acom	10 mm	Craniotomy, hematoma evacuation and clipping, (6 days)	Uneventful recovery, no neurological deficits, GOS 5, mRS 1

Kim et al. [20]/2005	49	72/f	WFNS 2, GCS 14	CT scan, DSA	Yes	Yes	Rt	6 mm	8 mm	Lt distal ACA	—	Hematoma evacuation and immediate clipping (48 h)	Dysphasia, right hemiparesis, GOS 3, mRS 4

Kim et al. [20]/2005	50	42/m	WFNS 5, GCS 3, bilaterally fixed pupils	CT scan, DSA	Yes	—	Lt	6.5 mm	10 mm	Lt ICA-Pcom	—	Hematoma evacuation and immediate clipping (3 h)	Mild left-sided arm paresis, GOS 4 mRS 3

Marinelli et al. [21]/2005	51	62/f	WFNS 1, GCS 15, complete left third nerve palsy	CT scan, MRI, MRA, DSA	No	No	Lt	—	—	Lt ICA-Pcom	10 mm	Endovascular embolization	Full recovery of left third nerve palsy, GOS 5, mRS 1

Hori et al. [22]/2005	52	57/m	WFNS 2, GCS 13-14, incomplete right oculomotor palsy	CT scan, DSA	No	No	Rt	—	Moderate to marked	Rt MCA	1.5 mm	Hematoma evacuation and immediate clipping	Full recovery, GOS 5, mRS 1

Koerbel et al. [23]/2005	53	62/f	WFNS 4, GCS 10-11, rapid neurological deterioration	CT scan, DSA	No	No	Lt	—	Moderate to marked	Lt ICA-Pcom	5 mm	Hematoma evacuation followed by coiling	Returned to normal lifestyle, GOS 5, mRS 1

Westermaier et al. [4]/07	54	55/f	WFNS 5, GCS 6, anisocoria right	CT scan, DSA	Yes	Yes	Rt	—	—	Rt Acom	—	EVD coiling and hematoma evacuation (24 h)	No formal deficits, mobile for short distance, GOS 4, Barthel 70

Westermaier et al. [4]/07	55	56/f	WFNS 5, GCS 3, MI, bilaterally fixed pupils, cardiopulmonary unstable	CT scan, DSA	Yes	Yes	Rt	—	—	Rt MCA	Large	Repeated infusions of manitol, hematoma evacuation, and immediate clipping (24 h)	Simple communication, left hemiparesis, permanent care, GOS 3, Barthel 20

Westermaier et al. [4]/07	56	55/f	WFNS 5, GCS 3, dilation of the right pupil	CT scan, DSA	Yes	No	Rt	—	—	Rt ICA-Pcom	—	Immediate hematoma evacuation, EVD and delayed coiling (24 h)	Mild left hemiparesis, GOS 4, Barthel 70

Westermaier et al. [4]/07	57	55/f	WFNS 5, GCS < 6, anisocoria right	CT scan, DSA	Yes	No	Rt	—	—	Rt Acom	—	Immediate hematoma evacuation, EVD, and delayed coiling (24 h)	Full recovery, return to work, GOS 5, mRS 1

Westermaier et al. [4]/07	58	43/f	WFNS 5, bilaterally fixed and dilated pupils	CT scan	Yes	No	Lt	—	—	Lt ICA-Pcom	—	Hematoma evacuation followed by coiling	Rt hemiparesis using a wheelchair for longer distances, GOS 3, Barthel 70

Westermaier et al. [4]/07	59	54/f	WFNS 5, GCS 3, dilation of the right pupil, cardiac instability	CT scan, DSA	Yes	—	Rt	—	—	Rt Acom	—	EVD, delayed coiling (24 h), hematoma evacuation three weeks later (burr hole)	Not able to walk, dependent on permanent care, GOS 3, Barthel 0

Westermaier et al. [4]/07	60	42/f	WFNS 5, dilation of the right pupil	CT scan, DSA	Yes	—	Rt	—	—	Rt ICA-Pcom	—	EVD, hematoma evacuation, and immediate clipping	Returned to normal lifestyle, GOS 5, Barthel 100

Westermaier et al. [4]/07	61	55/f	WFNS 5, bilaterally fixed pupils, cyanotic and hypoxic	CT scan	Yes	Yes	Rt	5 mm	4 mm	Rt MCA	14 mm	No therapy as a result of prolonged hypoxia before admission	Deceased, GOS 1, mRS 6

Gilad et al. [24]/2007	62	47/m	WFNS 1, GCS 15, partial left sixth cranial nerve palsy	CT scan, MRI, MRA, DSA	No	No	Tentorium midline	—	—	Intrasellar Acom	13 mm	Coil embolization alone	Uneventful, no neurological deficits, GOS 5, mRS 1

Suhara et al. [25]/2008	63	27/f	WFNS 4, GCS 8	CT scan, DSA	No	No	Rt	—	—	Lt Pcal (ACA)	7 mm	Craniectomy, immediate hematoma evacuation, and delayed clipping (5 days)	Uneventful recovery, no neurological deficits, GOS 5, mRS 1

Nishikawa et al. [26]/2009	64	45/m	WFNS 5, GCS 5, dilated slowly reacting pupils	CT scan, MRI, MRA	No	Yes	Bilateral	—	Moderate to marked	Lt ICA	—	Emergency hematoma evacuation, and clipping	Deceased (cerebral herniation 6 days after admission), GOS 1, mRS 6

Kocak et al. [27]/09	65	68/f	WFNS 5, GCS 6	CT scan, DSA	Yes	No		—	—	Rt ICA bifurcation	—	Patient died during resuscitation	Deceased, GOS 1, mRS 6

Kocak et al. [27]/09	66	53/m	WFNS 2, GCS 14	CT scan, DSA	Yes	No		—	—	Lt Pcom	—	Craniotomy, hematoma evacuation, and immediate clipping	Good recovery, GOS 5, mRs 1

Kocak et al. [27]/09	67	48/f	WFNS 3, GCS 10	CT scan, DSA (after hematoma evacuation)	Yes	No		—	Moderate to marked	Rt Pcom	—	Craniotomy and immediate hematoma evacuation, delayed clipping (6 days)	Severe disability, GOS 3, mRS 5

Kocak et al. [27]/09	68	63/f	WFNS 1, GCS 15	CT scan, DSA	Yes	No		—	—	Lt MCA	—	Craniotomy, hematoma evacuation, and immediate clipping	Good recovery, GOS 5, mRs 1

Kocak et al. [27]/09	69	51/f	WFNS 2, GCS 14	CT scan, DSA	Yes	No		—	—	Acom	—	Craniotomy, SDH evacuation, clipping	Good recovery, GOS 5, mRs 1

Kocak et al. [27]/09	70	72/f	WFNS 4, GCS 8	CT scan, DSA	Yes	Yes		—	Moderate to marked	Rt MCA	—	Craniotomy, hematoma evacuation (aSDH + ICH) and immediate clipping	Deceased, GOS 1, mRS 6

Kocak et al. [27]/09	71	56/f	WFNS 4, GCS 7	CT scan, DSA	Yes	Yes		—	Moderate to marked	Rt MCA	—	Craniotomy, hematoma evacuation (aSDH + ICH), and immediate clipping (6 h)	Deceased, GOS 1, mRS 6

Kocak et al. [27]/09	72	67/m	WFNS 5, GCS 5	CT scan, DSA (after hematoma evacuation)	Yes	No		—	Moderate to marked	Rt Pcom	—	Craniotomy and immediate hematoma evacuation, delayed clipping (8 days)	Severe disability, GOS 3, mRS 5

Kocak et al. [27]/09	73	47/f	WFNS 1, GCS 15	CT scan, CTA, DSA	No	No		—	—	Acom	—	Craniotomy, hematoma evacuation, and immediate clipping	Good recovery, GOS 5, mRs 1

Kocak et al. [27]/09	74	57/f	WFNS 3, GCS 13	CT scan, CTA, DSA	Yes	No		—	—	Lt Pcom	—	Craniotomy, hematoma evacuation, and immediate clipping	Good recovery, GOS 5, mRs 1

Kocak et al. [27]/09	75	46/f	WFNS 4, GCS 12	CT scan, CTA, DSA	Yes	No		—	—	Rt Pcom	—	Craniotomy, hematoma evacuation, and immediate clipping	Severe disability, GOS 3, mRS 5

Marbacher et al. [2]/10	76	44/f	WFNS 5, GCS 3, bilaterally fixed pupils	CT scan, DSA	Yes	No	Rt	15 mm	10 mm	Rt Pcal (ACA)	5 mm	Craniectomy, hematoma evacuation (4 h), and delayed clipping	Full recovery, mild cognitive deficits, GOS 5, mRS 1

Marbacher et al. [2]/10	77	50/f	WFNS 3, GCS 13, mild left-sided hemiparesis	CT scan, CTA	Yes	Yes	Rt	9 mm	23 mm	Rt MCA	11 mm	Craniectomy, hematoma evacuation (12 h), and delayed coiling	Mild left-sided arm paresis, GOS 4, mRS 2

Marbacher et al. [2]/10	78	39/m	WFNS 5, GCS 4, bilaterally fixed pupils	CT scan, CTA	Yes	No	Rt	10 mm	14 mm	Rt ICA-Pcom	5 mm	EVD, craniectomy, hematoma evacuation, and immediate clipping (18 h)	Residual left-sided hemiparesis, GOS 4, mRS 2

Marbacher et al. [2]/10	79	58/f	WFNS 5, GCS 5, dilation of the right pupil	CT scan, CTA	Yes	Yes	Rt	5 mm	4 mm	Rt MCA	14 mm	Craniectomy, hematoma evacuation, and immediate clipping (3 h)	Full recovery, mild cognitive deficits, GOS 5, mRS 1

Marbacher et al. [2]/10	80	45/f	WFNS 5, GCS 4, dilation of the right pupil	CT scan, DSA	Yes	No	Rt	20 mm	18 mm	Rt ICA-Pcom	7 mm	Craniectomy, hematoma evacuation, and immediate clipping (2 h)	Gait ataxia, GOS 4, mRS 3

Marbacher et al. [2]/10	81	68/f	WFNS 1, GCS 15, right oculomotor paresis	CT scan, CTA	Yes	No	Rt	10 mm	6 mm	Rt Distal ICA-Pcom	2 mm	Craniotomy, hematoma evacuation, and immediate clipping (6 h)	Full recovery, no symptoms at all, GOS 5, mRS 0

Marbacher et al. [2]/10	82	27/f	WFNS 5, GCS 3, bilaterally fixed mydriasis, unstable cardiopulmonary condition	CT scan, DSA	Yes	No	Rt	10 mm	7 mm	Rt Pcal (ACA)	12 mm	Craniectomy, hematoma evacuation (1 h)	Deceased, GOS 1, mRS 6

*Summary (characteristics) of 82 cases from 20 clinical case series or case reports of aneurysmal acute subdural hematomas. Abbreviations: SAH = subarachnoid hemorrhage; ICH = intracerebral hemorrhage; aSDH = acute subdural hematoma; MLS = midline shift; mm = millimeter; f = female; m = male; WFNS = World Federation of Neurological Surgeons; GCS = Glasgow Coma Scale; CT = computed tomography; Rt = right; Lt = left; mRS = modified Rankin Score; GOS = Glasgow Outcome Scale; FU = followup; NOS = not otherwise specified; Barthel = Barthel Index; DSA = digital subtraction angiography; MRI = magnetic resonance imaging; MRA = magnetic resonance angiography; MCA = middle cerebral artery; CTA = CT angiography; ICA = internal carotid artery; Pcom = posterior communicating artery; Acom = anterior communicating artery; ACA = anterior cerebral artery; Pcal = pericallosal artery; EVD = external ventricular drainage; MI = myocardial infarction.

**Table 3:** Outcome stratified according to therapeutic strategies*.

Patients presenting with rapidly deteriorating neurological condition				Patients presenting without rapidly deteriorating neurological condition			
Urgent intervention (<24 h)		Delayed intervention (>24 h)		Urgent intervention (<24 h)		Delayed intervention (>24 h)	
Outcome	*n* (%)	Outcome	*n* (%)	Outcome	*n* (%)	Outcome	*n* (%)
GOS 5 + 4	23 (64%)	GOS 5 + 4	6 (25%)	GOS 5 + 4	10 (100%)	GOS 5 + 4	5 (100%)
GOS 3 + 2	5 (14%)	GOS 3 + 2	2 (8%)	GOS 3 + 2	0 (0%)	GOS 3 + 2	0 (0%)
GOS 1	8 (22%)	GOS 1	16 (67%)	GOS 1	0 (0%)	GOS 1	0 (0%)

*Abbreviations: GOS = Glasgow Outcome Scale.
